# Creatinine-Based Formulae Poorly Match in the Classification of Hypofiltration or Hyperfiltration in a General Population of Adolescents: A Retrospective Analysis of a Cross-Sectional Study

**DOI:** 10.3389/fped.2021.719997

**Published:** 2021-10-28

**Authors:** Katarína Šebeková, Radana Gurecká, Ĺubomíra Tóthová, Ĺudmila Podracká

**Affiliations:** ^1^Faculty of Medicine, Institute of Molecular BioMedicine, Comenius University, Bratislava, Slovakia; ^2^Faculty of Medicine, Institute of Medical Physics, Biophysics, Informatics and Telemedicine, Comenius University, Bratislava, Slovakia; ^3^Department of Pediatrics, Faculty of Medicine, Comenius University, Bratislava, Slovakia; ^4^Department of Pediatrics, The National Institute of Children's Health, Bratislava, Slovakia

**Keywords:** kidney function, children, creatinine, adolescents, cardiometabolic risk (factors), estimated glomerular filtration rate (eGFR)

## Abstract

Pediatric formulae to estimate glomerular filtration rate (eGFR) give a broad range of values. Their consistency in assigning the subjects as hypofiltrating or hyperfiltrating is unknown. In 1993 apparently healthy adolescents (53.4% females) aged 14–17 years, we investigated the concordance of six creatinine-based formulae in the classification of the subjects into ≤ 5th or ≥95th percentile of eGFR, and the between-groups difference in the prevalence of cardiometabolic risk factors. Mean eGFR varied between 77 and 121 mL/min/1.73 m^2^. Arbitrary setting of hypofiltration or hyperfiltration to 5% returned 46 males and 53 females. At least one formula classified 89 males and 99 females as hypofiltrating and 105 males and 114 females as hyperfiltrating. All six formulae concordantly classified 15 males and 17 females as hypofiltrating and 9 and 14, respectively, as hyperfiltrating. Pairwise, formulae consistently classified hypofiltration in 42–87% of subjects with hyperfiltration in 28–94%. According to two out of the six formulae, hyperfiltration was associated with an increased prevalence of obesity and obesity-associated comorbidities. Hypofiltrating subjects did not manifest chronic kidney disease–associated comorbidities. Further studies in different populations of healthy adolescents are needed before it is possible to conclude which creatinine-based formula is appropriate for the classification of hypofiltration and hyperfiltration in nonclinical cohorts.

## Introduction

The worldwide increasing prevalence of hypertension and obesity in juveniles may predispose them to a rise in the manifestation of chronic kidney disease. Obesity-associated hyperfiltration is an antecedent of future chronic kidney disease (1, 2). Adolescent hypertension doubles the risk of end-stage renal disease regardless of the severity of hypertension and overweight (3). This urges the need for effective assessment of renal function in the general population of adolescents.

Serum creatinine–based estimated glomerular filtration rate (eGFR) is a widely used marker of renal function in both clinics and epidemiological studies. Despite that it is imperfect, it is generally employed because more accurate approaches for direct GFR assessment—renal and plasma clearance methods—can only be performed in specialized centers (4, 5). Urinary measurement of creatinine clearance requires the active cooperation of the subject in the accurate collection of urine over 24 h. The more accurate cystatin C-based estimation of GFR (4, 6, 7) is several times more expensive than the creatinine-based one. Thus, neither of these approaches is feasible in general practice or epidemiological studies. Formulae estimating GFR in adults, such as the MDRD or CKD-EPI equations (8, 9) do not apply to children and adolescents (10–12).

Derivation of universal pediatric equations faces several problems, such as sex differences in the growth spurt and muscle mass gain (4, 5, 13, 14). Currently, several equations derived from pediatric patients with renal disease or a general population of healthy subjects are available, but none of them ideally reflects measured GFR (4, 5). Studies in the general population of children and adolescents and pediatric patients with type 1 diabetes (T1D) comprehensively document the disparities and errors between measured and estimated GFR and show that different equations to estimate GFR return values in a broad range (13–16). However, it remains unclear whether different formulae consistently categorize adolescents into the lower and upper tail of eGFR distribution. Concordance in assigning is of clinical importance as a manifestation of low or high eGFR requires further diagnostic steps. To these points, we compared serum creatinine–based eGFR values obtained by six different equations in a large cohort of apparently healthy adolescents. We anticipated that the highest correlation across eGFR ranges as well as the highest consistency in assigning subjects into the tails of eGFR distribution, would be between pairs of equations derived from the same population. We also studied whether individuals with eGFR ≤ 5th percentile present morbidities that are commonly associated with decreased renal function and whether those displaying eGFR ≥ 95th percentile present obesity and obesity-associated risk markers.

## Subjects and Methods

This is a retrospective analysis of the data obtained in the cross-sectional study “Respect for Health.” The survey was launched in cooperation between the two local health authorities—the Department of Health of Bratislava Self-Governing Region and the Regional Public Health Authority of the Slovak Republic in Bratislava—and aimed to characterize the cardiometabolic health status of students attending public secondary schools in the Bratislava Region. Data were collected between November 2011 and December 2012. Acute illness, pregnancy, or lactation in females were exclusion criteria. Complete data on anthropometry and blood chemistry were obtained from 2960 students aged 12–23 years. For the current analysis, we extracted data on 14–17-year-old White Caucasian adolescents of Central European descent (*n* = 1,993; 53.4% females).

The study was conducted according to the Declaration of Helsinki after the approval of the protocol by the Ethics Committee of the Bratislava Self-Governing Region. The decision to participate was voluntary. Signed informed consent was obtained from parents or legal guardians of participants.

### Measurements

The study protocol has been explained in detail previously (17). Briefly, height, body weight, and blood pressure (BP) measurements were performed directly at high schools by trained staff, according to standard protocols (17). Body mass index (BMI) and waist-to-height ratio (WHtR) were calculated.

At appointed health centers, blood samples were collected after overnight fasting. Serum concentrations of glucose, high-density lipoprotein cholesterol (HDL-C), triacylglycerols, high-sensitivity C-reactive protein (CRP), insulin, and uric acid were analyzed in a central laboratory using standard analytical methods (ADVIA 2400 analyzer, Siemens, Erlangen, Germany). Serum creatinine was analyzed *via* a compensated, rate-blanked Jaffé reaction with an isotope dilution mass spectrometry (IDMS)–traceable calibrator (National Institute of Standards and technology, SRM 967). In spot urine, albumin (turbidimetrically) and creatinine concentrations were determined, and urinary albumin-to-creatinine ratio (ACR) was calculated.

### Estimation of Glomerular Filtration Rate

Serum creatinine–derived eGFR was estimated using six formulae, i.e., the Schwartz–Lyon formula (18), the formula of Léger et al. (19), the revised Lund–Malmö (LM) formula (20), the Lund–Malmö formula with lean body mass extension (LM-LBM) (21), and the full-age spectrum with Q-age (FAS-QA) or with Q-height extension (FAS-QH) (15), as follows:

**Schwartz–Lyon formula** **(****18****)**


$$\begin{array}{l} \;=k*\left(height\left[cm\right]/sCrea\left[\mu mol/L\right]\right)\\ k=\text{36}.\text{5 in males aged}>\text{13}\;\text{years}.\\ k=\text{32}.\text{5 in}\;\text{others} \end{array}$$

**Léger's formula** **(****19****)**


$$=56.7*weight\left[kg\right]+0.142*heigh{\left[cm\right]}^{2}/sCrea\left[\mu mol/L\right]\;$$

**LM formula** **(****20****)**


$$\begin{array}{l} =e^{X-0.0158*age+0.438*ln(age)}\;\\ \text{Males}:\\ \text{X=2}.\text{56+0}.\text{00968}*(\text{180-sCrea}[\mu \text{mol/L}]),\text{if sCrea}\\ \leq \text{180}\;\mu \text{mol/L}\\ \text{X=2}.\text{56-0}.\text{926}*\text{ln}(\text{sCrea}[\mu \text{mol/L}]\text{/180}),\text{if sCrea}\\ \geq \text{180}\mu \text{mol/L}\\ \text{Females}:\\ \text{X=2}.\text{50+0}.\text{0121}*(\text{150-sCrea}[\mu \text{mol/L}]),\text{if sCrea}\\ \leq \text{150}\;\mu \text{mol/L}\\ \text{X=2}.\text{50-0}.\text{926}*\text{ln}(\text{sCrea}[\mu \text{mol/L}]\text{/150}),\text{if sCrea}\\ \geq \text{150}\;\mu \text{mol/L} \end{array}$$

**LM-LBM formula** **(****20****)**


$$\begin{array}{l} =e^{X-0.00587*age+0.00977^{*}LBM}\;\\ \text{X=4}.\text{95-0}.\text{0110}*\text{sCrea}[\mu \text{mol/L}],\text{if}\;\text{sCrea}\leq \text{150}\mu \text{mol/L}\\ \text{X=8}.\text{58+0}.\text{0005}*\text{sCrea}[\mu \text{mol/L}]\text{-1}.\text{08}*\text{ln}(\text{sCrea}[\mu \text{mol/L}]),\\ \text{if}\;\text{sCrea}\geq \text{150}\;\mu \text{mol/L} \end{array}$$

LBM:


$$\begin{array}{l} \text{ Males}:\text{LBM=1}.\text{10}*\text{weight}[\text{kg}]\\ \;-\text{120}*{\left(\text{weight}[\text{kg}]\text{/height}\;[\text{cm}]\right)}^{\text{2}}\\ \text{Females}:\text{LBM=1}.\text{07}*\text{weight}[\text{kg}]\\ \;\;\;\;\;\;\;\;\;\;\;\;\;\;\;\;\;\;\;\;-\text{148}*{\left(\text{weight}[\text{kg}]\text{/height}\;[\text{cm}]\right)}^{\text{2}} \end{array}$$

**FAS-QH** **(****15****)**


$$\begin{array}{l} \;=107.3/\left(sCrea\left[mg/dL\right]/Q\right)\\ \text{Q}=\text{3}.\text{94}-\text{13}.\text{4}*\left(\text{height}[\text{m}]\right)\text{+17}.\text{6}*{\left(\text{height}[\text{m}]\right)}^{\text{2}}\\ \;-\text{9}.\text{84}*{\left(\text{height}[\text{m}]\right)}^{\text{3}}\text{+2}.\text{04}*{\left(\text{height}\;[\text{m}]\right)}^{\text{4}} \end{array}$$

**FAS-QA** **(****15****)**


$$\begin{array}{l} \;=107.3/(sCrea[mg/dL]/Q)\\ \text{ Males}:\text{Q}=0.21+0.057*\text{age}-0.0075*{\text{age}}^{2}+0.00064*{\text{age}}^{3}\\ \;-0.000016^{*}{\text{age}}^{4}\\ \text{Females}:\text{Q}=0.23+0.034*\text{age}-0.0018*{\text{age}}^{2}+0.00017*{\text{age}}^{3}\\ \;-0.0000051*{\text{age}}^{4} \end{array}$$

eGFR is expressed in mL/min/1.73 m^2^, height is expressed in centimeters except for the FAS-QH formula, in which it is expressed in meters, sCrea: serum creatinine concentration is expressed in micromoles per liter (μmol/L) except for the FAS formulae, in which it is expressed in mg/dL (conversion factor: 88.42), age is expressed in years, body weight in kilograms, Ln: natural logarithm, e: the base of the natural logarithm.

Arbitrarily, hypofiltration and hyperfiltration were set at the formula- and sex-specific ≤ 5th and ≥95th eGFR percentiles, respectively. We also used the conventional cutoffs: ≤ 75 mL/min/1.73 m^2^ and ≥135 mL/min/1.73 m^2^ as recommended for adolescents (22, 23).

### Definition of Cardiometabolic Risk Factors

The presence of general overweight and obesity was classified using the international age- and sex-specific cutoff points (24); that of central obesity is WHtR ≥ 0.5 (25). The presence of elevated systolic BP (SBP ≥ 130 mmHg) or diastolic BP (DBP ≥ 85 mmHg), elevated triacylglycerols (TAG ≥ 1.7 mmol/L), low HDL-C (males: <1.03 mmol/L, females: <1.29 mmol/L), increased atherogenic index of plasma [=log (TAG/HDL-C)] ≥ 0.11 (26), fasting glycemia (≥5.6 mmol/L), elevated uric acid levels (≥340 μmol/L in females, ≥420 μmol/L in males), the concentration of fasting insulin ≥20 μIU/mL (27), or CRP > 3 mg/L (28) were considered as markers of increased cardiometabolic risk. Microalbuminuria was classified as ACR 2.5–25.0 mg/mmol in males and 3.5–35.0 mg/mmol in females (29).

### Statistics

Data characterizing the cohort are given as mean ± SD for not normally distributed data as the median (interquartile range). Body weight, height, and BMI *z*-scores were calculated using the age- and sex-specific national reference data from 2001 (30). The descriptive characteristic of eGFR obtained by different formulae is given as the mean, SD, and 5th and 95th percentiles separately for males and females. The prevalence of subjects presenting eGFR under or above the conventional cutoffs for hypofiltration and hyperfiltration in adolescents is given as count and percentage. Between-sex differences in GFR estimated by different formulae were compared using the two-sided unpaired Student's *t*-test. Mutual regressions of eGFR values obtained by six equations were expressed as coefficients of determination (*R*^2^). The agreement of different formulae in returning individuals' eGFR within the lower or upper tail of eGFR distribution, i.e., ≤ 5th or ≥95th percentile, was examined either as a match of designation by all six equations or pair-wise. Fisher's exact test was used to compare the prevalence of cardiometabolic risk factors between subjects within the lower and the upper tail of each eGFR distribution. Data are presented as means or as counts. A *P* < 0.05 was considered statistically significant. Analyses were performed by using the SPSS v.16 for Windows software (SPSS Inc., Chicago, IL, USA).

## Results

Cohort characteristics are given in Table 1. Males and females were of similar age and had mean serum creatinine concentrations of 74.8 ± 13.2 and 60.9 ± 7.6 μmol/L, respectively. Forty-three (4.5%) males and 13 (1.2%) females presented serum creatinine levels above their age-specific upper reference limit defined by Pottel et al. (23).

**Table 1:** Characteristics of the studied population.

	**Males**	**Females**
*n*	929	1,046
Age, years	16.1 ± 0.8	16.2 ± 0.8
Height, cm	178.8 ± 7.0	165.7 ± 6.2
Height *Z*-score	0.15 ± 1.02	0.01 ± 1.00
Body weight, kg	73.1 ± 14.2	59.9 ± 10.2
Body weight *Z*-score	0.62 ± 1.30	0.32 ± 1.13
BMI, kg/m^2^	22.8 ± 3.9	21.8 ± 3.4
BMI *Z*-score	0.61 ± 1.28	0.34 ± 1.10
WHtR	0.44 ± 0.05	0.43 ± 0.05
SBP, mm Hg	122 ± 12	107 ± 9
FPG, mmol/L	4.9 ± 0.5	4.7 ± 0.8
FPI, μUI/mL	9.4 (7.9; 13.0)	9.9 (7.4; 13.4)
HDL-C, mmol/L	1.25 ± 0.23	1.48 ± 0.28
TAG, mmol/L	0.85 ± 0.43	0.85 ± 0.37
AIP	−0.20 ± 0.22	−0.27 ± 0.20
Uric acid, μmol/L	355 ± 60	262 ± 52
ACR, mg/mmol	0.4 (0.2; 0.6)	0.5 (0.3; 0.8)
CRP, mg/L	0.4 (0.2; 1.0)	0.4 (0.2; 1.0)

*BMI, body mass index; WHtR, waist-to-height ratio; SBP, systolic blood pressure; FPG, fasting plasma glucose; FPI, fasting plasma insulin; HDL-C, high-density lipoprotein cholesterol; TAG, triacylglycerols; AIP, atherogenic index of plasma; ACR, urinary albumin-to-creatinine ratio; CRP, C-reactive protein, data given as mean±standard deviation or as median (interquartile range), Z-scores were calculated based on the national sex- and age-specific standards*.

### Glomerular Filtration Rate Estimated via Different Formulae

Descriptive characteristics of data on eGFR obtained by each formula are given in Table 2. In both sexes, the LM equation yielded the lowest mean eGFR values and the lowest cutoffs for the 5th and 95th percentiles. The highest values were obtained using Léger's formula. The difference between means returned by Léger's vs. LM formula reached about 41 mL/min/1.73 m^2^ in males and 45 mL/min/1.73 m^2^ in females. At the 5th percentile, it corresponded to about 29 mL/min/1.73 m^2^ and 33 mL/min/1.73 m^2^, respectively; at the 95th, to about 56 mL/min/1.73 m^2^ in males and 62 mL/min/1.73 m^2^ in females.

**Table 2:** Descriptive characteristics of estimated glomerular filtration rate returned by six creatinine-based formulae in males and females.

**eGFR formula**	**Sch-L**	**Léger**	**LM**	**LM-LBM**	**FAS-QH**	**FAS-QA**
**Males**						
Mean	89.2	118.6	78.0	95.4	112.5	101.1
Standard deviation	12.9	20.8	11.8	12.0	22.0	14.1
5th percentile	71.1	90.6	61.5	78.0	83.2	81.5
95th percentile	111.7	155.1	98.8	114.1	153.6	125.8
≤ 75 mL/min/1.73 m^2^, *n* (%)	98 (10.5)	5 (0.5)	398 (42.8)	26 (2.8)	16 (1.7)	11 (1.2)
≥135 mL/min/1.73 m^2^, *n* (%)	4 (0.4)	175 (18.8)	1 (0.1)	1 (0.1)	135 (14.5)	14 (1.5)
**Females**						
Mean	89.8	121.6	76.5	101.8	107.5	105.2
Standard deviation	11.3	18.6	10.0	9.1	16.0	13.4
*P* (vs. males)	0.276	**0.001**	**0.002**	** <0.001**	** <0.001**	** <0.001**
5th percentile	72.7	94.7	61.8	86.9	84.5	85.4
95th percentile	108.9	155.3	93.4	116.8	137.9	128.7
≤ 75 mL/min/1.73 m^2^, *n* (%)	80 (7.5)	0	499 (46.9)	3 (0.3)	6 (0.6)	4 (0.4)
≥135 mL/min/1.73 m^2^, *n* (%)	1 (0.1)	229 (21.5)	1 (0.1)	1 (0.1)	64 (6.3)	21 (2.1)

*eGFR, estimated glomerular filtration rate; Sch-L, Schwartz-Lyon formula; LM, Lund-Malmö formula; LM-LBM, Lund-Malmö formula with lean body mass extension; FAS-QH, the full-age spectrum formula with Q-height extension; FAS-QA, the full-age spectrum formula with Q-age extension, significant p is given in bold*.

Employing the LM equation, 43% of males presented eGFR <75 mL/min/1.73 m^2^; with the Schwartz–Lyon formula assigned to this category, about 11% of males; with the other four equations, the prevalence was <3% (Table 2). In females, the prevalence of eGFR <75 mL/min/1.73 m^2^ reached about 47, 8, and 0–1%, respectively. Using Léger's and the FAS-QH formulae, the prevalence of eGFR ≥ 135 mL/min/1.73 m^2^ reached about 19 and 15%, respectively, in males and 22 and 6% in females. The remaining four formulae returned prevalence between 0.1 and 2% in both sexes.

Mean eGFR calculated using the Schwartz–Lyon formula did not differ between the sexes. Léger's, LM-LBM, and FAS-QA equations gave higher mean eGFR in females compared with males; the opposite was observed using the LM and FAS-QH equations (Table 2).

**Figure 1:**
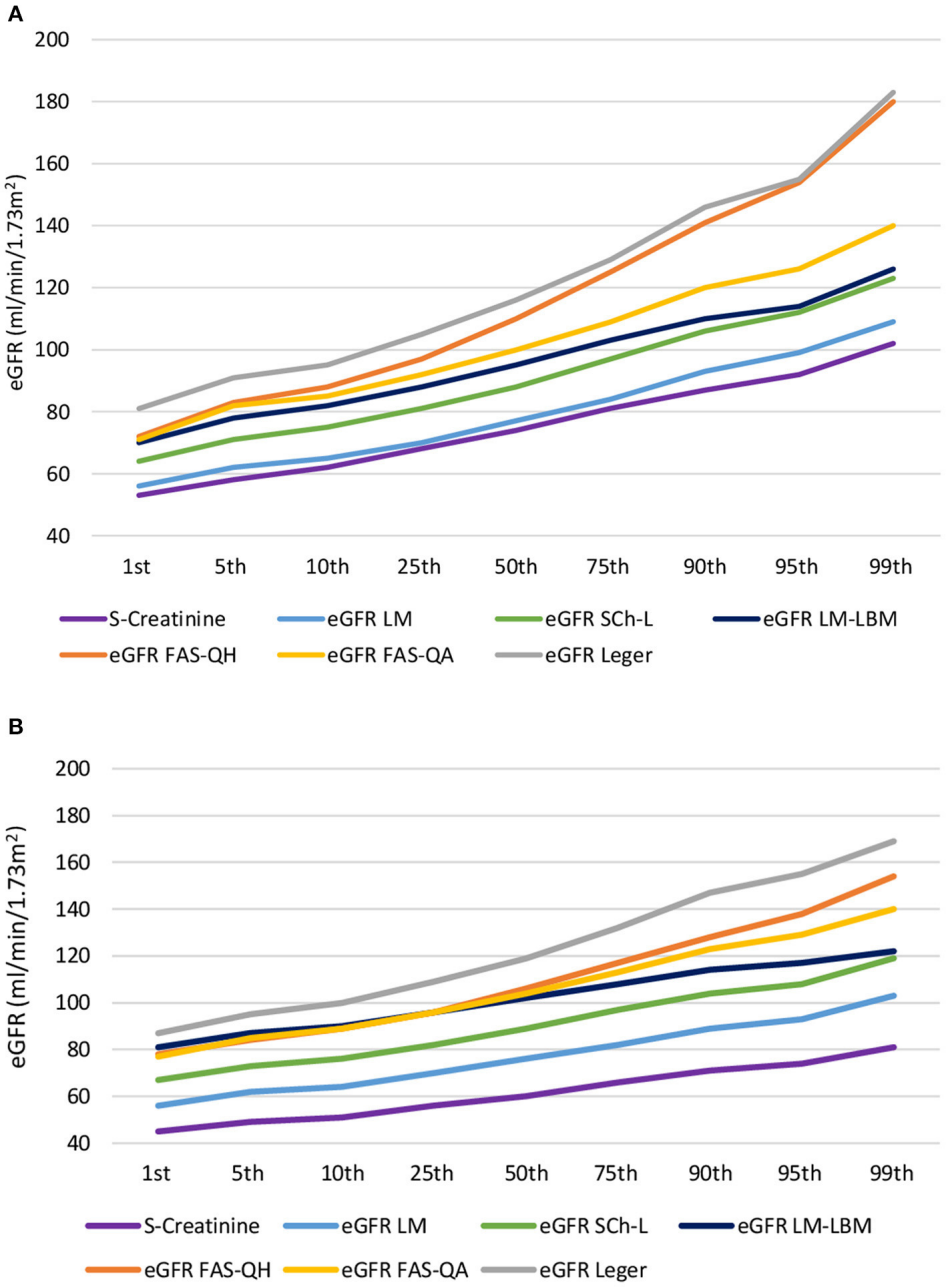
Chart flow of serum creatinine concentration and estimated glomerular filtration rate values returned by six formulae across the 1st to 99th percentile in **(A)** males and **(B)** females. eGFR, estimated glomerular filtration rate; Sch-L, Schwartz-Lyon formula; LM, Lund-Malmö formula; LM-LBM, Lund-Malmö formula with lean body mass extension; FAS-QH, the full-age spectrum formula with Q-height extension; FAS-QA the full-age spectrum formula with Q-age extension.

For a better understanding of the differences, chart flows of serum creatinine concentration and eGFR values returned by six formulae across the 1st to 99th percentile were plotted (Figures 1A,B). Among two formulae derived from different cohorts of children with chronic kidney disease in both sexes, Léger's equation returned higher eGFR values compared with the Schwartz–Lyon equation. As for formulae derived from the same populations, in both sexes, the LM-LBM equation returned higher eGFR values compared with the LM formula. From eGFR > 82 mL/min/1.73 m^2^ in males and about 104 mL/min/1.73 m^2^ in females, FAS-QH gave higher eGFR values compared with FAS-QA, particularly in males (Figures 1A,B).

In males, LM-LBM, FAS-QA, and FAS-QH equations returned similar eGFR values at the 1st percentile (Figure 1A). Thereafter, the slope of the LM-LBM equation flattened, and from the 95th percentile onward (about 113 mL/min/1.73 m^2^), LM-LBM eGFR values copied those returned by the Schwartz–Lyon equation. Up to the 5th percentile, both FAS formulae gave almost identical eGFR values. Although the slope of the FAS-QA equation showed minor variations across the percentiles, that of the FAS-QH equation rose more steeply, matching the values returned by Léger's formula at the 95th and 99th percentiles (i.e., ≥155 mL/min/1.73 m^2^).

In females, eGFR lines of both FAS and the LM-LBM formulae started to deviate above the 50th percentile, corresponding to an eGFR of 102–106 mL/min/1.73 m^2^. Thereafter, the slope of the LM-LBM formula flattened, reaching the 99th percentile value similar to that returned by the Schwartz–Lyon equation, and that of the FAS-QH equation rose more rapidly than that of FAS-QA, but in contrast to males, it did not reach values returned by Léger's equation at the 99th percentile (Figure 1B).

In both sexes, coefficients of determination between LM and Schwartz–Lyon, LM and FAS-QA, and LM-LBM and Léger's equations were >0.9 (Table 3). Schwartz–Lyon showed a good correlation with LM-LBM or FAS-QA formulae both in males and females, and in females, it reached a value >0.8 also vs. the FAS-QH formula. In both sexes, equations derived from the same population, i.e., FAS-QH vs. FAS-QA or LM vs. LM-LBM, showed poor association (Table 3).

**Table 3:** Coefficients of determination between estimated glomerular filtration rate returned by six formulae in males (right upper corner) and females (lower left corner).

	**Sch-L**	**Léger**	**LM**	**LM-LBM**	**FAS-QH**	**FAS-QA**
Sch-L	–	0.691	0.925	0.846	0.637	0.841
Léger	0.712	–	0.534	0.912	0.679	0.507
LM	0.910	0.546	–	0.736	0.377	0.916
LM-LBM	0.830	0.929	0.709	–	0.643	0.674
FAS-QH	0.815	0.731	0.551	0.736	–	0.507
FAS-QA	0.872	0.523	0.976	0.669	0.523	–

*Sch-L, Schwartz-Lyon formula; LM, Lund-Malmö formula; LM-LBM, Lund-Malmö formula with lean body mass extension; FAS-QH, the full-age spectrum formula with Q-height extension; FAS-QA, the full-age spectrum formula with Q-age extension*.

### Consistency of Six Formulae in the Classification of Subjects as Hypofiltrating or Hyperfiltrating

Arbitrary setting of the prevalence to 5% returned 46 males and 53 females.

At least one formula classified 89 males as hypofiltrating. Of those, six formulae matched in the classification of 15 males (17% out of 89); 10 (11%) subjects were concordantly classified by any five as well as by any four formulae out of six; three equations consistently identified 12 males (13%), two formulae achieved concordance in 19 males (21%), and in 23 males (26%) only one out of six formulae indicated hypofiltration.

As for the concordance of classification between the pairs of equations, the Schwartz–Lyon vs. LM-LBM and LM vs. FAS-QA showed the highest (80%, both), and two FAS equations the lowest (43%) concordance in sorting males to the lower tail of eGFR distribution (Table 4). For example, of 46 males classified as hypofiltrating by the Schwartz–Lyon formula, the LM-LBM equation classified as hypofiltrating 37, and 43% concordance observed for FAS formulae means that the second formula denotes as hypofiltrating 20 out of 46 subjects in the lower tail of the distribution of the first formula.

**Table 4:** Consistency between pairs of equations in assigning males and females into the ≤ 5th percentile (the upper right corner) and into the ≥95th percentile (the lower left corner) of each eGFR distribution.

	**Sch-L**	**Léger**	**LM**	**LM-LBM**	**FAS-QH**	**FAS-QA**
**Males**						
Sch-L	–	61%	78%	80%	63%	67%
Léger	48%	–	48%	72%	72%	43%
LM	83%	39%	–	70%	46%	80%
LM-LBM	59%	85%	48%	–	67%	65%
FAS-QH	46%	48%	30%	52%	–	43%
FAS-QA	67%	37%	76%	44%	28%	–
**Females**						
Sch-L	–	62%	83%	74%	71%	69%
Léger	57%	–	49%	83%	67%	42%
LM	68%	40%	–	62%	54%	87%
LM-LBM	57%	94%	40%	–	67%	53%
FAS-QH	70%	53%	45%	55%	–	42%
FAS-QA	70%	42%	79%	42%	47%	–

*eGFR, estimated glomerular filtration rate; Sch-L, Schwartz-Lyon formula; LM, Lund-Malmö formula; LM-LBM, Lund-Malmö formula with lean body mass extension; FAS-QH, the full-age spectrum formula with Q-height extension; FAS-QA, the full-age spectrum formula with Q-age extension*.

Among females, 99 were classified as presenting hypofiltration by at least one formula. Of them, 17 were concordantly classified as hypofiltrating by all six formulae, any five or any four formulae matched in the classification of 12 females, three formulae concordantly classified 11, and any two equations reached concordance in 26 individuals. Twenty-one females were classified as hypofiltrating by any one of the six formulae. This corresponded to 17, 12, 12, 11, 26, and 21% of 99 subjects.

In females, FAS-QA vs. LM, LM-LBM vs. Léger's, and LM vs. Schwartz–Lyon equations yielded the highest (87–83%) matches in the classification of hypofiltration, and the poorest concordance (42%) was between the FAS formulae (Table 4). For example, FAS-QA and LM formulae indicating 87% matching concordantly assigned as hypofiltrating 46 of 53 females, in the case of the two FAS formulae, it was 22 out of 53.

One hundred five males were classified as hyperfiltrating by at least one formula. Six formulae matched in the classification of nine males (about 9% out of 105); eight (8%) subjects were concordantly classified by any five, 12 (11%) by any four formulae out of six; three equations consistently identified 18 males (17%), two formulae achieved concordance in 22 males (21%), and in 36 males (34%), only one out of six formulae indicated hyperfiltration. Léger's and LM-LBM, and SchL and LM formulae reached a consistency of 85 and 83%, respectively, in assigning males as hyperfiltrating (Table 4). The poorest concordance (28%) was revealed between the two FAS formulae.

Among females, 114 were classified as presenting hyperfiltration by at least one formula. All six, and any five, four, three, and two formulae concordantly classified 14, 10, 12, 13, and 33 females, respectively; corresponding to 12, 9, 11, 11, and 29% out of 114 subjects. One formula indicated hyperfiltration in 32 females (28%). As in males, Léger's and LM-LBM formulae showed the highest matching (94%) in the classification of hyperfiltration, followed by LM with FAS-QA (79%) (Table 4). The poorest agreement (40%) was observed between LM and LBM as well as between LM and Léger's equations.

### Comparison of Mean Age, Height, and the Prevalence of Cardiometabolic Risk Markers Among Adolescents With eGFR ≤ 5th vs. ≥95th Percentile by Various Pediatric GFR Estimating Equations

Using all but FAS equations, males classified as hypofiltrating were significantly younger (by 4–11 months) compared with their peers assigned to the upper tail of eGFR distribution (Table 5). With the height-independent equations, males presenting in the eGFR ≤ 5th percentile were taller. The opposite was observed employing the height-dependent formulae; with the FAS-QH equation, the means differed by almost 18 cm (Table 5).

**Table 5:** Mean estimated glomerular filtration rate, mean age, and mean height of males and females assigned by six formulae to the lower (≤ 5th) and the upper (≥95th) tail of each distribution.

**Formula**	**eGFR, mL/min/1.73 m** ^**2**^			**Age, years**			**Height, cm**		
	**eGFR perc**.		***p***	**eGFR perc**.		**p**	**eGFR perc**.		***p***
	**≤5th**	**≥95th**		**≤5th**	**≥95th**		**≤5th**	**≥95th**	
**Males**									
Sch-L	65	120	** <0.001**	16.4	15.5	** <0.001**	176.3	178.3	0.215
Léger	82	172	** <0.001**	16.3	15.8	**0.013**	173.1	183.3	** <0.001**
LM	56	106	** <0.001**	16.4	15.5.	** <0.001**	181.3	175.0	** <0.001**
LM-LBM	70	121	** <0.001**	16.4	16.7	** <0.001**	176.4	182.7	** <0.001**
FAS-QH	75	168	** <0.001**	16.3	15.9	0.055	171.2	189.1	** <0.001**
FAS-QA	74	134	** <0.001**	16.0	16.0	1.000	181.3	175.7	**0.001**
**Females**									
Sch-L	82	133	** <0.001**	16.4	16.2	0.101	164.5	168.8	** <0.001**
Léger	85	127	** <0.001**	16.3	16.1	0.155	161.9	169.9	** <0.001**
LM	81	135	** <0.001**	16.2	16.1	0.337	167.3	163.6	**0.002**
LM-LBM	84	127	** <0.001**	16.4	16.0	**0.016**	163.9	169.7	** <0.001**
FAS-QH	86	125	** <0.001**	16.3	16.0	0.075	160.6	174.4	** <0.001**
FAS-QA	81	136	** <0.001**	15.9	16.4	**0.002**	168.0	164.5	**0.003**

*eGFR, estimated glomerular filtration rate; SCh-L, Schwartz-Lyon formula; LM, Lund-Malmö formula; LM-LBM, Lund-Malmö formula with lean body mass extension; FAS-QH, the full-age spectrum formula with Q-height extension; FAS-QA, the full-age spectrum formula with Q-age extension, significant p is given in bold*.

Using the Léger's and LM-LBM equations, the prevalence of central obesity, general overweight/obesity, elevated BP, low HDL-C, elevated atherogenic index, and CRP > 3 mg/L was significantly higher in males at the upper tail of eGFR distribution vs. the lower tail (Table 6). The prevalence of general obesity was also higher by Schwartz–Lyon and FAS-QA formulae. All but FAS-QA equations indicated a higher prevalence of elevated fasting insulin in subjects with eGFR ≥ 95th percentile. The prevalence of elevated triacylglycerols, uric acid, and that of microalbuminuria did not differ between the groups (Table 6).

**Table 6:** The prevalence of cardiometabolic risk factors in males with estimated glomerular filtration rate ≤ 5th vs. that of ≥95th according to six creatinine-based equations.

**Formula**	**WHtR** **≥** **0.5**			**BMI Ow/Ob OITF**			**BMI Ob IOTF**			**SBP** **≥** **130 or DBP** **≥** **85 mm Hg**			**Glycemia** **≥** **5.6 mmol/L**			**Insulin** **≥** **20** **μUI/mL**		
	**eGFR perc**.		***p***	**eGFR perc**.		***p***	**eGFR perc**.		***p***	**eGFR perc**.		***p***	**eGFR perc**.		***p***	**eGFR perc**.		***p***
	**≤5th**	**≥95th**		**≤5th**	**≥95th**		**≤5th**	**≥95th**		**≤5th**	**≥95th**		**≤5th**	**≥95th**		**≤5th**	**≥95th**	
Sch-L	4	10	0.145	15	18	0.664	1	8	**0.030**	10	9	1.000	2	2	1.000	2	11	**0.014**
Léger	1	29	** <0.001**	6	39	** <0.001**	0	25	** <0.001**	5	19	**0.002**	3	2	1.000	3	21	** <0.001**
LM	4	10	0.089	15	16	0.828	2	8	0.050	12	7	0.304	3	1	0.617	3	10	**0.040**
LM-LBM	3	22	** <0.001**	10	35	** <0.001**	1	18	** <0.001**	9	17	0.104	3	2	1.000	3	17	**0.001**
FAS-QH	4	8	0.345	12	18	0.266	1	7	0.059	9	20	**0.024**	3	2	1.000	1	11	**0.004**
FAS-QA	3	10	0.069	13	14	1.000	1	8	**0.030**	11	10	1.000	2	2	1.000	3	10	0.069
**Formula**	**HDL-C** **<** **1.29 mmol/L**			**TAG** **≥** **1.7 mmol/L**			**AIP** **≥** **0.11**			**UA** **≥** **340** **μmol/L**			**ACR** **≥** **3.5 mg/mmol**			**CRP** **>** **3 mg/L**		
	**eGFR perc**		***p***	**eGFR perc**		***p***	**eGFR perc**		***p***	**eGFR perc**		***p***	**eGFR perc**		***p***	**eGFR perc**		***p***
	**≤5th**	**≥95th**		**≤5th**	**≥95th**		**≤5th**	**≥95th**		**≤5th**	**≥95th**		**≤5th**	**≥95th**		**≤5th**	**≥95th**	
Sch-L	8	8	1.000	0	3	0.242	2	5	0.434	7	3	0.315	2	2	1.000	2	8	0.090
Léger	8	19	**0.021**	1	6	0.111	1	11	**0.004**	5	11	0.168	1	0	1.000	1	9	**0.015**
LM	9	8	1.000	2	3	0.677	3	5	0.486	8	2	0.091	2	3	0.677	1	6	0.059
LM-LBM	9	16	0.159	2	6	0.267	2	10	**0.027**	6	9	0.574	2	0	0.495	1	8	**0.030**
FAS-QH	8	10	0.793	1	2	1.000	2	4	0.677	9	4	0.231	1	2	1.000	2	6	0.267
FAS-QA	10	8	0.793	2	3	1.000	2	5	0.434	10	3	0.069	1	2	1.000	2	8	0.090

*The prevalence is given as the number of males presenting the risk factor out of 46/group*.

*Formulae: Sch-L, Schwartz-Lyon; LM, Lund-Malmo; LM-LMB, Lund-Malmo with lean body mass; FAS-QH, Full-age Spectrum with Q-height; FAS-QA, Full-age Spectrum with Q-age*.

*WHtR, waist-to-height ratio; BMI, body mass index; ow/ob, overweight/obese; OITF, The International Obesity Taskforce; SBP, systolic blood pressure; DBP, diastolic blood pressure; HDL-C, high-density lipoprotein cholesterol; eGFR, estimated glomerular filtration rate; TAG, triacylglycerols; AIP, atherogenic index of plasma; ACR, UA, uric acid; urinary albumin-to-creatinine ratio; CRP, C-reactive protein, significant p is given in bold*.

With the LM-LBM formula, females at the upper tail of eGFR distribution were about 5 months younger than their peers at the lower tail, and the FAS-QA equation returned the opposite (Table 5). As in males, LM and FAS-QA equations indicated that females at the upper tail of distribution were shorter compared with their hypofiltrating peers, and the opposite was observed employing the height-dependent formulae. With the FAS-QH equation, the height difference reached about 14 cm.

Using the FAS-QA formula, females manifesting eGFR ≤ 5th percentile more likely suffered from hyperuricemia than their counterparts with eGFR ≥ 95th percentile (Table 7). Léger's and LM-LBM equations indicated a higher prevalence of central obesity, general overweight/obesity or obesity, elevated fasting insulin, and CRP levels and low HDL-C in females at the upper tails of eGFR distributions compared with those on the lower ones. None of the equations indicated a difference in the prevalence of elevated BP, hypertriacylglycerolemia, elevated atherogenic index, or microalbuminuria.

**Table 7:** The prevalence of cardiometabolic risk factors in females with estimated glomerular filtration rate ≤ 5th vs. that of ≥95th according to six creatinine-based equations.

**Formula**	**WHtR** **≥** **0.5**			**BMI Ow/Ob OITF**			**BMI Ob IOTF**			**SBP** **≥** **130 or DBP** **≥** **85 mm Hg**			**Glycemia** **≥** **5.6 mmol/L**			**Insulin** **≥** **20** **μUI/mL**		
	**eGFR perc**.		***p***	**eGFR perc**.		***p***	**eGFR perc**.		***p***	**eGFR perc**.		***p***	**eGFR perc**.		***p***	**eGFR perc**.		***p***
	**≤5th**	**≥95th**		**≤5th**	**≥95th**		**≤5th**	**≥95th**		**≤5th**	**≥95th**		**≤5th**	**≥95th**		**≤5th**	**≥95th**	
Sch-L	1	4	0.363	9	8	0.797	0	2	0.495	3	1	0.363	2	2	1.000	0	4	0.118
Leger	1	17	** <0.001**	1	30	** <0.001**	0	11	**0.001**	4	3	1.000	2	2	1.000	1	9	**0.016**
LM	1	7	0.060	6	11	0.290	0	2	0.495	4	0	0.118	1	2	1.000	0	4	0.118
LM-LBM	1	16	** <0.001**	2	29	** <0.001**	0	10	**0.001**	3	3	1.000	2	2	1.000	0	9	**0.003**
FAS-QH	2	4	0.697	10	6	0.286	0	1	1.000	3	2	0.675	1	2	1.000	0	3	0.243
FAS-QA	0	5	0.057	6	9	0.579	0	3	0.243	4	1	0.363	1	1	1.000	0	4	0.118
**Formula**	**HDL-C** **<** **1.29 mmol/L**			**TAG** **≥** **1.7 mmol/L**			**AIP** **≥** **0.11**			**UA** **≥** **340** **μmol/L**			**ACR** **≥** **3.5 mg/mmol**			**CRP** **>** **3 mg/L**		
	**eGFR perc**		***p***	**eGFR perc**		***p***	**eGFR perc**		***p***	**eGFR perc**		***p***	**eGFR perc**		***p***	**eGFR perc**		***p***
	**≤5th**	**≥95th**		**≤5th**	**≥95th**		**≤5th**	**≥95th**		**≤5th**	**≥95th**		**≤5th**	**≥95th**		**≤5th**	**≥95th**	
Sch-L	9	17	0.131	4	1	0.205	2	2	1.000	8	2	0.052	2	2	1.000	5	8	0.555
Leger	5	22	** <0.001**	2	3	1.000	1	5	0.205	7	5	0.761	4	0	0.118	5	14	**0.041**
LM	9	18	0.073	3	1	0.618	2	1	1.000	9	3	0.123	2	2	1.000	4	7	0.526
LM-LBM	5	20	**0.001**	2	3	1.000	1	5	0.205	8	5	0.555	3	0	0.243	4	13	**0.032**
FAS-QH	7	15	0.094	2	1	0.614	0	2	0.495	4	2	0.433	2	2	1.000	2	6	0.271
FAS-QA	8	17	0.066	3	1	0.618	2	1	1.000	10	2	**0.028**	2	1	1.000	4	7	0.526

*The prevalence is given as the number of females presenting the risk factor out of 53/group*.

*Formulae: Sch-L, Schwartz-Lyon; LM, Lund-Malmo; LM-LMB, Lund-Malmo with lean body mass; FAS-QH, Full-age Spectrum with Q-height; FAS-QA, Full-age Spectrum with Q-age*.

*WHtR, waist-to-height ratio; BMI, body mass index; ow/ob, overweight/obese; OITF, The International Obesity Taskforce; SBP, systolic blood pressure; DBP, diastolic blood pressure; HDL-C, high-density lipoprotein cholesterol; eGFR, estimated glomerular filtration rate; TAG, triacylglycerols; AIP, atherogenic index of plasma; ACR, UA, uric acid; urinary albumin-to-creatinine ratio; CRP, C-reactive protein, significant p is given in bold*.

## Discussion

We explored the agreement between six creatinine-based formulae for estimation of GFR in a cohort of apparently healthy adolescents, intending to find out which equations match the best in the classification of hyperfiltration and hypofiltration. As expected, different formulae returned a broad range of eGFR values. Importantly, there was not a simple numerical shift in eGFR values returned by different formulae. Across eGFR percentiles, the trajectories returned by different formulae largely were not parallel, and the differences between eGFR values rose more steeply in males than females. We also revealed a substantial mismatch between formulae in assigning the subjects into the upper and lower tails of eGFR distributions. In females, two equations—Léger's and LM-LBM formulae—showed a clinically acceptable match in assigning the subjects into both the lower and upper tails of eGFR distribution. However, adolescents of both sexes classified by these two equations as hyperfiltrating manifested a higher prevalence of obesity and obesity-associated comorbidities compared with their hypofiltrating peers. Adolescents assigned into the lower eGFR tails did not present an increased prevalence of comorbid conditions consistent with CKD except for a moderately higher prevalence of hyperuricemia in females with GFR ≤ 5th percentile according to the FAS-QA formula.

In line with former studies in healthy or T1D adolescents (13, 14, 16) and in our study, different pediatric formulae returned diverse mean eGFR values. Five out of six equations indicated sex differences in mean eGFR that were inconsistent regarding whether mean eGFR was higher in males or females. The sex differences did not match completely the sex differences reported by Boettcher et al. (16) for a large group of children and adolescents with T1D. The difference between the highest and the lowest mean eGFR in our study was similar to that reported for 12–17-year-old U.S. adolescents (43 mL/min/1.73 m^2^) (13) but was higher than the difference of about 16 mL/min/1.73 m^2^ in males and 20 mL/min/1.73 m^2^ in females observed in 1- to <18-year-old T1D patients (16).

Differences among serum creatinine–based formulae derived to estimate GFR in the pediatric population stem from the differences in variables included in the equations (i.e., age, height, body weight, and sex), mathematical forms, and coefficients calculated to fit best the source data, e.g., the populations that were used to derive the equations. Among six formulae compared in our study, data on patients with kidney diseases served as sources in the construction of four of them. Schwartz–Lyon (18) and Léger's (19) formulae were derived from pediatric patients with CKD, or CKD and kidney-transplanted patients, respectively. The LM and LM-LBM equations were developed from data on adults mostly with renal disease (21), and later they were evaluated in a pediatric cohort consisting mainly of subjects with suspected or confirmed CKD (31). It is suggested that, as in adults, also in children and adolescents equations developed in populations with decreased GFR underestimate GFR among those without kidney disease (13). However, in our cohort, Léger's formula consistently overestimated eGFR compared with values returned by FAS-QA and FAS-QH equations in both sexes, and in females, LM-LBM gave slightly higher eGFR means than both FAS formulae between the 1st to about the 25th percentile. It is assumed that variations in creatininemia in patients with CKD are more likely to reflect changes in GFR. Muscle mass, growth, or protein intake are more important determinants of serum creatinine levels in subjects without CKD. In our study, the LM equation gave the lowest eGFR among the six formulae throughout the whole range of creatinine values in both sexes and, thus, the highest prevalence (>40%) of subjects with hypofiltration (eGFR <75 mL/min/1.73 m^2^), followed by the Schwartz–Lyon formula. This is in line with data on 1- to <18-year-old males with T1D although, in females, the Schwartz–Lyon formula gave lower means compared with the LM equation (16). As in the study of Boettcher et al. (16), the introduction of a lean body mass component into the LM equation returned higher mean eGFR compared with the LM equation. However, with GFR above 110 mL/min/1.73 m^2^, the overestimation of eGFR by the LM-LBM equation compared with LM and Schwartz–Lyon formulae gradually diminished (Figures 1A,B). Although both LM equations were derived from the same population, their trajectories across eGFR percentiles were not parallel. Léger's (19) formula returned the highest eGFR values throughout the whole serum creatinine range and showed the steepest rise across the percentiles. The difference between the 1st and 99th eGFR percentile reached a difference of 100 mL/min/1.73 m^2^ in males and 80 mL/min/1.73 m^2^ in females. With the other formulae derived from patients with CKD, differences between the 1st and the 99th eGFR percentile varied between 50 and 60 mL/min/1.73 m^2^ in males and 40 and 50 mL/min/1.73 m^2^ in females. Nonetheless, for Léger's equation, the study in U.S. adolescents reported a similar difference in eGFR (about 120 mL/min/1.73 m^2^) across the percentiles (13) as observed by us. Of note, creatininemia ranged about 45–100 μmol/L in both studies.

The FAS-QA and FAS-QH formulae intended to provide equations for all ages without the discontinuity between pediatric and adult equations based on Belgian data on 0.1–20-year-old healthy subjects (15). The FAS-QA formula enables eGFR calculation in case the anthropometric data is not available. In line with findings in adolescents with T1D (16), in our females, both FAS equations returned similar mean eGFR although, in males, mean FAS-QH eGFR was slightly higher than that given by FAS-QA. In both sexes, the FAS-QA formula overestimated eGFR in comparison with the LM and Schwartz–Lyon formulae, but the rise in eGFR across the percentiles was similar. FAS-QH formula–derived eGFR rose sharply across the percentiles—particularly in males, in whom the difference across the percentiles corresponded to that observed for Léger's equation, and at the upper end of the distribution, two formulae returned almost identical eGFR values. This finding is surprising as it has been assumed that eGFR values returned by equations based on data from patients with renal disease differ from those derived from healthy subjects (5), and that the FAS-QH equation outperforms the other height-dependent formulae in healthy adolescents (15). Likewise, the discrepancy between FAS-QA and FAS-QH formulae, particularly in our males, is surprising. It suggests that a single normalization constant derived from a population of healthy Belgian adolescents might not be representative of other European adolescents.

Theoretically, numerically different eGFR values returned by different formulae would not be confounding if reported along with age- and sex-specific reference ranges. Knowing which formula had been used and what the particular cutoffs are, the clinician or epidemiologist provided with a simple eGFR value would be able to judge whether an individual should be subjected to further diagnostic steps. However, this assumption would be plausible only if different formulae consistently classify the same subject as hypofiltrating or hyperfiltrating. This requirement is important for both general practice and epidemiological studies as subjects with low or high eGFR should be referred for further examination to confirm or reject kidney disease. Our data suggest that, in apparently healthy adolescents, a conventional cutoff limit for hypofiltration or hyperfiltration cannot be universally applied. The LM formula–derived eGFR yielded extremely high frequencies of eGFR ≤ 75 mL/min/1.73 m^2^ in both sexes, and the Schwartz–Lyon formula probably also overestimated the prevalence in our males. Applying the other formulae, the frequencies of abnormal eGFR were low and corresponded with those reported for the general population of adolescents in other studies (13, 23). On the other hand, Léger's and FAS-QH yielded a very high prevalence of eGFR ≥ 135 mL/min/1.73 m^2^ in both sexes, and Sch-L, LM, and LM-LBM hardly identified a single hyperfiltrating individual.

Similarly, a concordance of six formulae in assigning the same subject to the lowest or the highest 5% of eGFR distribution was poor. In both sexes, among 15 possible pairs between six formulae, the coefficient of determination ≥90% was revealed for three pairs (LM vs. Sch-L or FAS-QA and Léger vs. LM-LBM). However, overall correlations seemed not to be sensitive enough to detect variations occurring at the extremities of the distribution. Although in females these three pairs of equations also showed acceptable matching (≥80%) for assigning the probands into the lowest tail of eGFR distribution; in males, it was only LM vs. FAS-QA and, additionally, the Sch-L vs. LM-LBM pair. The differences in eGFR calculated by different formulae increase with higher eGFR (13, 14). However, we show that, even the values at the 5th percentile are diverse: the height-independent formulae (LM and FAS-QA) showing an acceptable matching for classification of hypofiltration in both sexes, return at the 5th percentile values differing by ≥20 mL/min/1.73 m^2^. Regarding hyperfiltration, LM vs. Sch-L and Léger's formula vs. the LM-LMB showed ≥80% matching in adolescent males; in females, only the latter pair returned acceptable concordance. Our data do not support our hypothesis on the consistent classification of subjects to the lower or the upper tail of eGFR distribution by formulae derived from the same populations. As in apparently healthy adolescents, eGFR results indicating hypofiltration or hyperfiltration estimated by different creatinine-based formulae are equivocal. The interpretation of the results must be done with caution. This underscores the importance of clinical decision making, which includes multiple factors in addition to eGFR (32). Our data suggest that the question on the reliability of different formulae in the estimation of GFR (33) is not restricted to adults.

Except for the fact that both height-independent formulae assigned shorter individuals into the lower tail of eGFR distributions and that the FAS-QA equation indicated a moderately higher prevalence of hyperuricemia in females with eGFR ≤ 5th percentile compared with those within the upper 5%, no group of participants with eGFR ≤ 5th percentile showed an increased prevalence of other conditions consistent with CKD. These findings are in line with those reported for U.S. adolescents in the lower ranges of eGFR (13). On the other hand, using Léger's and LM-LBM formulae, hyperfiltrating subjects of both sexes presented a higher prevalence of general and central obesity, dyslipidemia, fasting hyperinsulinemia, and CRP > 3 mg/L compared with their hypofiltrating peers. The U.S. study in nondiabetic adolescents also reported an association of glomerular hyperfiltration with hypertriacylglycerolemia and lower insulin sensitivity (34).

The strengths of our study are a reasonably large sample of White Caucasian adolescents for whom data were gathered within one school year, that morphometry was performed by trained staff according to the same protocol, and serum samples were analyzed in a central laboratory referencing creatinine assay to IDMS standards. We do not have data on renal or plasma creatinine clearance or the measured GFR; thus, we only could assess agreement between the formulae rather than which formula is most accurate. Observed associations might be biased by the potential participation of close relatives. We have no data on factors potentially influencing eGFR, such as birth weight, prematurity, history of former urinary tract infections, lifestyle and genetic factors, or family history of CKD. Our results are based on a single measurement, and we did not follow renal function status over time. There are limitations of generalizing our findings to populations with different epidemiological, anthropometric, or clinical characteristics.

In conclusion, our data show that relationships between eGFR values returned by pediatric formulae are largely lax, and the concordance of the equations in assigning apparently healthy adolescents as hypofiltrating or hyperfiltrating is generally poor. We did not follow renal function status over time, and to our knowledge, there is no data from other populations to compare potential discrepancies in the assignment of adolescents to tails of eGFR distributions. Thus, it remains questionable which eGFR formula should be used in adolescents to screen for abnormal renal function in general practice or epidemiological studies. This presents an opportunity for future studies in longitudinal cohorts.

## Data Availability Statement

The raw data supporting the conclusions of this article will be made available by the authors, without undue reservation.

## Ethics Statement

The studies involving human participants were reviewed and approved by the Ethics Committee of Bratislava Self-Governing Region. Written informed consent to participate in this study was provided by the participants' legal guardian/next of kin.

## Author Contributions

KŠ and RG: drafting of the manuscript, statistical analysis, and had full access to all of the data in the study and take responsibility for the integrity of the data and the accuracy of the data analysis. KŠ and ĹP: study concept and design. KŠ, RG, and ĹT: acquisition of data. KS: study supervision. All authors: critical revision of the manuscript for important intellectual content, analysis, or interpretation of data.

## Funding

This work was supported by grants from The Slovak Research and Development Agency No. APVV-0447-12, and APVV-18-0287; The Scientific Grant Agency of the Ministry of Education, Science, Research and Sport of the Slovak Republic and the Slovak Academy of Sciences No. 1/0637/13; and the Bratislava Self-governing Region.

## Conflict of Interest

The authors declare that the research was conducted in the absence of any commercial or financial relationships that could be construed as a potential conflict of interest.

## Publisher's Note

All claims expressed in this article are solely those of the authors and do not necessarily represent those of their affiliated organizations, or those of the publisher, the editors and the reviewers. Any product that may be evaluated in this article, or claim that may be made by its manufacturer, is not guaranteed or endorsed by the publisher.
