# Supplementary figures and images for: Creatinine-Based Formulae Poorly Match in the Classification of Hypofiltration or Hyperfiltration in a General Population of Adolescents: A Retrospective Analysis of a Cross-Sectional Study
Source: Front Pediatr. 2021 Oct 28;9:719997. doi: 10.3389/fped.2021.719997 (PMC8581240; doi:10.3389/fped.2021.719997)

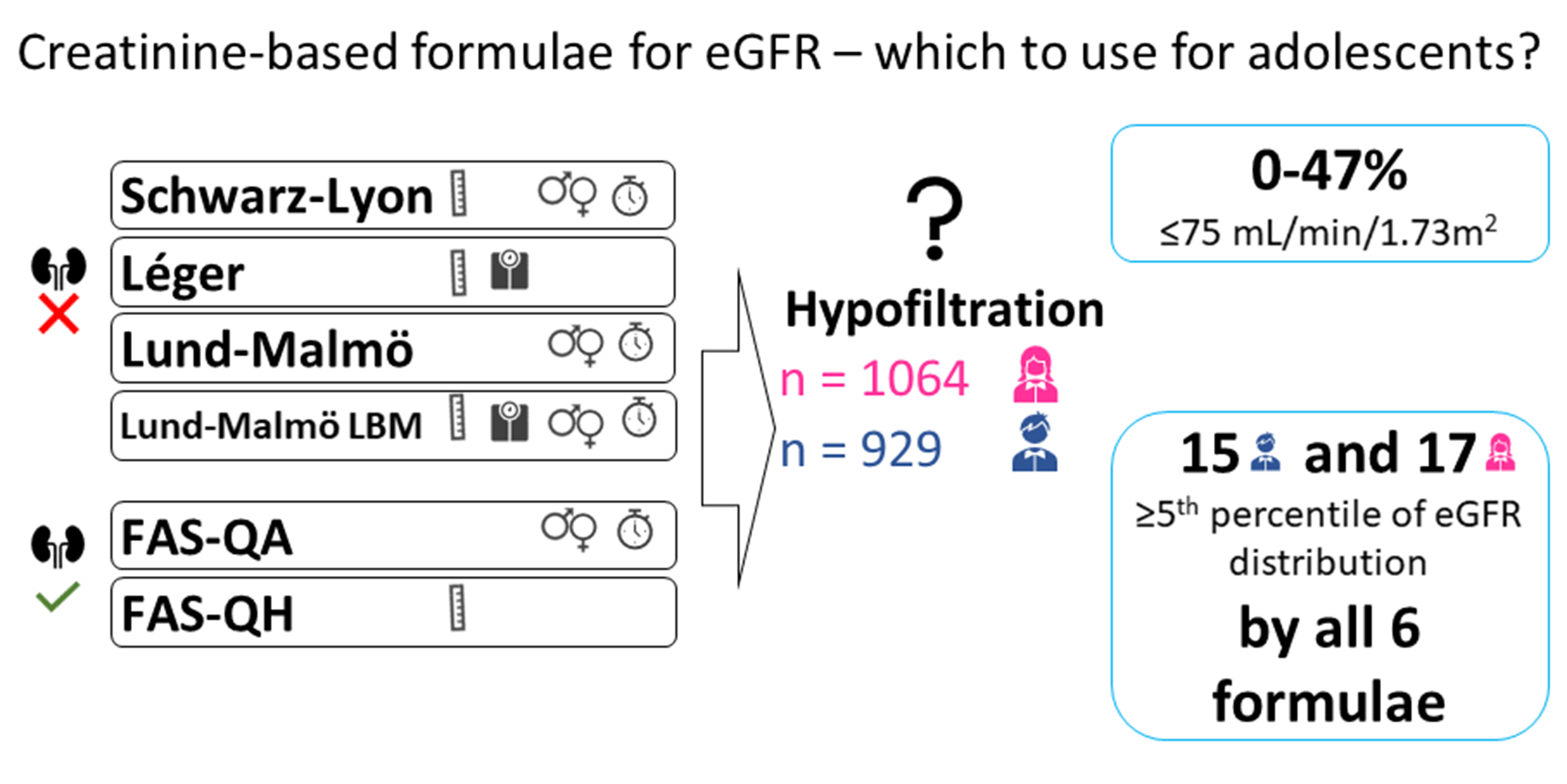

Supplement: Supplementary file 1 [file Image_1.TIF]
